# Why Do Student Perceptions of Teachers’ Condemning of the Bullying and Empathy-Raising Lead to Intention to Stop Bullying? The Role of Emotions

**DOI:** 10.1007/s11121-025-01839-2

**Published:** 2025-09-12

**Authors:** Eerika Johander, Tiina Turunen, Claire F. Garandeau, Christina Salmivalli

**Affiliations:** 1 INVEST Flagship Research Center/Department of Psychology and Speech-Language Pathology, University of Turku, Turku, Finland; 2 Shandong Normal University, Jinan, China

**Keywords:** Bullying, Targeted interventions, Teacher messages, Guilt, Shame, Sadness

## Abstract

Two strategies used by teachers in targeted antibullying interventions, condemning of the bullying behavior and empathy-raising, have been shown to positively predict bullies’ intention to stop bullying. However, the mechanism through which they work remains unknown. We tested whether moral emotions (guilt and shame), and empathic emotion (sadness) mediated the effects of students’ perceptions of teachers’ condemning and empathy-raising messages on their intention to stop bullying. A normative sample of 277 seventh-grade students (*M*_age_ = 12.93, SD = 0.49; 47% female) was asked to imagine having bullied a peer and being invited to a discussion with a teacher. They saw a video vignette with one of three messages: condemning, empathy-raising or a combination of both. Analyses revealed that the effects of perceived condemning of the bullying behavior on intention to stop were primarily mediated by feelings of guilt, while the effects of perceived empathy-raising were mediated by both sadness and guilt. Shame was not associated with intention to stop bullying and did not mediate the effects of the perceptions. These findings suggest that targeted anti-bullying interventions should aim to evoke guilt and sadness (or empathic concern) rather than shame.

## Introduction

Studies on teachers’ targeted interventions have focused on examining the effectiveness of different strategies that teachers might use when talking to students who have bullied others. Results from studies conducted in primary and secondary schools indicate that both condemning the behavior and trying to raise perpetrators’ empathy for their victimized peer can be effective in stopping bullying or reducing intention to continue bullying, although the interventions fail in almost 26% of cases (Garandeau et al., 2014, 2016; Johander et al., 2021, 2022). However, no study has investigated why these approaches work. To further enhance the effectiveness of targeted interventions, it is essential to identify these mechanisms.

Since emotions (or anticipated emotions) can shape behavior (see DeWall et al., 2016), and targeted interventions should logically elicit emotions (e.g., guilt) in students, one reason why teacher-delivered anti-bullying messages work may be by arousing specific emotions, which in turn affect the perpetrators’ decision to stop or pursue their behavior. Moral emotions such as guilt and shame, as well as emotions related to empathic concern, such as sadness, have the potential to motivate students to behave in prosocial ways (e.g., de Hooge et al., 2018; Eisenberg et al., 1989; Olthof, 2012). These emotions are likely elicited in intervention discussions, since condemning the behavior emphasizes how morally wrong bullying is, whereas attempts to arouse empathy should make the perpetrator understand the suffering their behavior is causing.

Most studies on the effects of targeted intervention strategies have considered the approach that teachers were instructed to use or reported using, rather than *how these approaches were perceived by the bullying students*. In order to find out through which emotions different interventions lead to students’ intention to stop bullying, it is relevant to examine the effects of the messages (condemning or empathy-raising) as they are perceived by the students themselves. Therefore, this study examines whether the effects of perceived condemning of bullying behavior and perceived empathy-arousal on students’ intention to stop bullying are mediated by self-evaluative moral emotions, namely guilt and shame (e.g., Tangney et al., 2007), or by emotions associated with empathic concern, such as sadness (e.g., Van der Graaff et al., 2016).

### Effectiveness of Confronting and Non-Confronting Approaches in Tackling Bullying

Studies on teachers’ targeted interventions have investigated two major approaches: a confronting and a non-confronting approach (e.g., Garandeau et al., 2014). The confronting approach focuses on condemning the bullying and setting clear limits for unacceptable behavior. In the discussion, perpetrator(s) are told that the school personnel know about their behavior, bullying is wrong, not tolerated, and must stop immediately (see Olweus, 1993). This is the most common approach used by teachers (Rigby & Bauman, 2010). The non-confronting approach, derived from the Method of Shared Concern (Pikas, 1989) and the Support Group Method (Robinson & Maines, 2008), is an indirect strategy aiming to arouse perpetrator(s) empathy for their victimized peer. Adults express their concern for the difficulties experienced by the victim without taking a stand on who is responsible. The goal is to get the perpetrator(s) to share the adult’s concern for the victimized peer’s situation and provide a solution to improve it. Similar strategies have been advocated in the bullying literature for decades; for instance, the Olweus Bullying Prevention Program (OBPP) has long emphasized the importance of clearly condemning bullying and setting firm behavioral boundaries (Olweus & Limber, 2010), while the Method of Shared Concern (Pikas, 1989) has empathy-raising as a central component.

To date, four studies have directly compared the effects of the confronting and non-confronting approaches. Two were conducted within the context of the randomized controlled trial (RCT) of the KiVa antibullying program in Finland, which includes both approaches, and investigated their short-term effects (Garandeau et al., 2014, 2016). One was carried out after the nationwide roll-out of the same program and investigated the effectiveness of different methods as evaluated by students and teachers retrospectively in the end of the school year (Johander et al., 2021), while another study utilized a video vignette experiment among a sample of adolescents (Johander et al., 2022). Both approaches were found to be equally effective in reducing bullying (as reported by students who had been victimized) in the short term (bullying had stopped or decreased in 97.7% of the cases; Garandeau et al., 2014) and in the longer term (bullying had stopped or decreased in 74% of the cases; Johander et al., 2021). Further, there is indication that combining the two approaches by both condemning the behavior and attempting to raise empathy for the victim is more likely to lead to intention to stop bullying among perpetrator(s) than using either of these strategies alone (Garandeau et al., 2016; Johander et al., 2022). Although individual characteristics such as empathy and callous-unemotional traits are known to influence the effectiveness of the methods (Johander et al., 2022), the psychological mechanisms behind the effects remain unknown. To better understand how youth engaging in bullying respond – psychologically – to interventions and to further improve these interventions, it is important to identify the emotions that they elicit and clarify whether each approach works by eliciting the same or different emotions.

### The Mediating Role of Emotions

Emotions serve as powerful motivators and regulators of our actions (e.g., Baumeister et al., 2007). Bullying, defined as intentional, repeated aggression towards a less powerful peer (Olweus, 1993), is an immoral behavior involving deliberate infliction of harm to others. Moral emotions (guilt and shame) and emotions related to empathic concern (sadness) are likely to motivate bullying students to change their behavior.

Guilt and shame are self-conscious moral emotions that involve negative self-evaluation and feelings of distress elicited by one’s perceived failures or transgressions (Tangney et al., 2007). Although guilt and shame have much in common and often co-occur (e.g., Hendriks et al., 2022), they are distinct emotions. Guilt is hypothesized to arise from recognition of one’s immoral action, whereas shame relates to feelings about one’s self-worth (Lewis, 1971, as cited in Tangney et al., 2007). Regarding their interpersonal consequences, guilt is often adaptive since it can motivate reparative behaviors (Roos et al., 2014; Schmader & Lickel, 2006). However, the interpersonal consequences of shame are less straightforward. Some studies suggest that shame is associated with avoidance behavior (Schmader & Lickel, 2006), aggression (Åslund et al., 2009) and decreases in prosocial behavior (Roos et al., 2014). Other studies suggest that shame can also lead to motivation to affiliate (de Hooge et al., 2018) and to reparative behaviors (de Hooge et al., 2010).

Sadness is an emotion characterized by unpleasant or negative valence and low arousal (Hartmann et al., 2023). It is often elicited by negative experiences, such as loss or failure (e.g., Shirai & Suzuki, 2017). Witnessing or learning about another person’s distress can induce empathic sadness (e.g., Stuijfzand et al., 2016), which is a form of empathic concern (Batson, 2023; Hall & Schwartz, 2019), an affective aspect of the broader concept of empathy – the ability to understand and share the feelings of another. Empathic concern includes a range of other-oriented emotions, such as feelings of sympathy, compassion and concern for the other (Batson, 2023; Hall & Schwartz, 2019). Importantly, sadness in response to another person’s suffering reflects an individual’s ability to emotionally resonate with others and has been used as an indicator of empathic sadness and concern in previous research (e.g., Eisenberg et al., 1988; Stuijfzand et al., 2016). Limited research has examined associations between situational or state empathic concern (as opposed to dispositional or trait empathy) and interpersonal behavior. However, in the few available studies, both state empathic sadness and concern have been positively associated with prosocial and reparative behaviors (Eisenberg et al., 1989; Schumann & Dragotta, 2021).

Although any kind of anti-bullying intervention conveys the message that bullying is not a desirable behavior, explicitly condemning it – as the confronting approach does – emphasizes that bullying is morally wrong, which is likely to evoke guilt (Tangney & Dearing, 2002). At the same time, such condemnation may lead some adolescents to perceive their actions as misaligned with social and moral standards, resulting in feelings of shame (Fessler, 2007). Moreover, although the intervention aims to condemn the behavior rather than the individual, some adolescents may internalize this condemnation as a reflection of their character, which could also evoke feelings of shame (Tangney & Dearing, 2002). The goal of the non-confronting approach, on the other hand, is to arouse empathy and concern in the perpetrator for the pain their actions have caused by describing what has been happening to the targeted student and how distressful the situation must be. The enhanced awareness of the suffering of the targeted student is likely to evoke sadness. Moreover, it has been proposed that guilt and empathy are linked (Tangney et al., 2007) and guilt can be an empathic response or arise from empathy through one's self-attribution as the cause of another's suffering (e.g., Baumeister et al., 1994). It is also possible that interventions arising empathy evoke moral emotions of guilt and shame as well, since guilt and shame often co-occur (e.g., Hendriks et al., 2022).

### Current Study

This study investigates how early adolescents’ perception of an antibullying message affects their intention to stop their (hypothetical) bullying. Students watched a video of an adult talking to them after they have supposedly bullied a peer, using one of three messages: condemning (as in the confronting approach), empathy-raising (as in the non-confronting approach), or a combination of both. In the condemning message, the adult explicitly states that they know the student has been involved in bullying perpetration, that bullying is strictly forbidden and must stop immediately. In the empathy-raising message, the adult does not mention that they know the student has been involved in bullying. Rather, they describe how they feel sad and concerned about the situation of the victimized student, what has been happening to them, and how it must feel awful to go to school when such things are happening. In the combined message, the adult conveys both the condemning and empathy-raising aspects, stating that bullying is strictly forbidden and must stop immediately while also expressing concern for the victimized student and their experience. After watching the video, participants responded to a questionnaire assessing the extent to which they felt that the adult had condemned their bullying behavior and tried to arouse their empathy for the victim. They were also asked to indicate how guilty, ashamed, and sad they would feel, and how likely they would be to stop their bullying behavior if their teacher would talk to them in this way.

First, we examine whether and how perceived condemning of the bullying and perceived empathy-arousal are associated with participants’ intention to stop bullying, hypothesizing that both are positively associated with intention to stop. Second, we examine whether guilt, shame, and sadness are associated with the intention to stop. We hypothesize positive effects of guilt and sadness, but we set no hypothesis regarding feeling ashamed due to inconsistencies in past findings. Third, we examine whether the perceived messages are associated with feelings of guilt, shame, and sadness. We hypothesize positive effects of condemning of the bullying behavior on guilt and shame, but not on sadness, and positive effects of perceived empathy-arousal on sadness and guilt, but not on shame. Finally, we examine whether the associations between each perceived message and intention to stop are mediated by feeling guilty, ashamed, or sad. We hypothesize that guilt mediates the association between perceived condemning of bullying behavior and intention to stop, and that sadness mediates the association between perceived empathy-raising and intention to stop. All other indirect effects are tested exploratively.

This study is conducted on a normative sample and therefore also includes students who are not particularly high in bullying behavior. However, a substantial number of students engage in bullying at some point in their lives (e.g., Modecki et al., 2014), and an even greater number participate in different roles, such as reinforcing the bullies’ behavior or joining in once the bullying has started (e.g., Salmivalli, 2010). Therefore, investigating students’ cognitive and emotional responses to antibullying messages in a normative sample is highly relevant.

## Method

### Procedure

A convenience sample of secondary schools (*n* = 3) and combined (both primary and secondary grades) schools (*n* = 4) in Finland participated in the data collection. Due to the COVID-19 pandemic and the subsequent school lockdown that took place during the spring of 2020, data was collected on three separate occasions: February 2020, May 2020, and September–October 2020. In February and September–October, the data was collected in schools by the first author and trained research assistants using pen-and-paper questionnaires. In May, it was collected online.

Principals of the participating schools were explained the study purpose and procedures and asked to invite all seventh-graders in their school to participate. Information about the study procedures and data protection were sent to the parents or guardians of the students, and they were asked for an informed consent for their child to participate. All students who returned a completed parental consent form participated in a token lottery (two movie tickets per classroom) regardless of whether their parents consented to their participation. Only students who received parental consent and provided their own assent took part in the study. The study was approved by the Ethics Committee for Human Sciences of the University of Turku.

Each classroom was assigned to one of the three conditions (condemning, empathy-raising, and combined message) except one particularly large classroom, where students were randomly divided across the three conditions. To make data collection easier for the schools, students from several classrooms assigned to the same condition (within the same school) were gathered together to participate in the same test group. Participants completed a questionnaire before and after watching the video where a teacher delivered one of the three anti-bullying messages. The survey filled out before watching the video included questions about participants’ age, gender, and bullying behavior. Prior to seeing the video and answering these questions, participants were shown a definition of bullying and asked to imagine that they had been involved in bullying a peer at school and the teacher had invited them to discuss the situation. They were instructed to listen attentively, since the video would be played only once. Each group saw one of the six videos (i.e., one of the three messages delivered by either a male or female teacher) together. Afterward, participants answered questions individually about their perception of the extent to which the teacher had condemned their bullying behavior or tried to arouse their empathy for the victim. They were also asked how guilty, ashamed, and sad they would feel, and how likely they would be to stop bullying after such a discussion, if it happened to them in real life.

### Participants

The sample included 295 students from 38 classrooms divided into 22 test groups (students who participated online were considered as one test group); 273 responded to the pen-and-paper questionnaires at school and 22 of them responded to the online questionnaire at home. Of these 22 students, nine reported that they had not watched the video before moving to the second part of the questionnaire and were excluded from further analyses. The online participants only saw messages presented by the female teacher. Among the 273 pen-and-paper participants, nine were excluded due to clearly patterned responses on the survey. The final sample consisted of 277 students (129 females, 147 males, one with missing information on gender; *M*age = 12.93, SD = 0.49) from 37 classrooms and 22 test groups. In a previous study using the same data, participants’ intention to stop bullying behavior was quite high (*M* = 4.15 on a scale from 0 to 5) across the different messages, right after hearing them (Johander et al., 2022).

### Measures

#### Intention to Stop Bullying Behavior

Six items assessed participants’ intention to stop bullying behavior: *If I had been in this situation and the teacher would have talked to me like this, (a) I would stop bullying the classmate; (b) it would be unlikely that I would bully others in the future because of what the teacher said to me; (c) I would not bully others anymore after this discussion; (d) I would probably continue bullying after this (reverse coded); (e) what the teacher said would very likely influence how I treat others in the future; and (f) the teacher’s words would have a strong impact on my behavior.* Responses were given on a 6-point scale ranging from 0 = strongly disagree to 5 = strongly agree. The reliability coefficient McDonald’s omega (see Hayes & Coutts, 2020) for these six questions was good (Ω =.84).

#### Perceived Condemning of the Bullying Behavior

Three items assessed the extent to which participants felt that the teacher had condemned their bullying behavior: *(a) the teacher clearly mentioned that I have behaved wrongly; (b) the teacher told me that he/she knew that I had been bullying my classmate and demanded that I stop; and (c) the teacher blamed me for the things that have happened.* Responses were given on a 6-point scale ranging from 0 = strongly disagree to 5 = strongly agree. The scores for the three items were averaged and the composite score had good reliability (Ω =.86).

#### Perceived Empathy-Raising

Four items assessed the extent to which participants felt that the teacher had tried to arouse their empathy towards the hypothetical victim: *(a) the teacher talked especially about how bad my classmate is feeling; (b) the teacher tried to make me understand how bad my classmate is feeling; (c) the teacher did not blame me, but wanted me to help the classmate who is having a difficult time; and (d) the teacher helped me to understand the difficult situation my classmate is in.* Responses were given on a 6-point scale ranging from 0 = strongly disagree to 5 = strongly agree. The scores for the four items were averaged and the composite score had good reliability (Ω =.79).

#### Guilt, Shame, and Sadness

One item assessed each of the three emotional reactions: *If I had been in this situation and the teacher would have talked to me like this, I would feel guilty/ashamed/sad.* Responses were given on a 6-point scale ranging from 0 = not at all to 5 = very much.

#### Control variables

Gender of the participant (0 = girl, 1 = boy), the teacher (0 = female, 1 = male), and self-reported frequency of bullying in real life were used as control variables in the analyses. The global single item of bullying from the revised Olweus’s Bully/Victim Questionnaire (Olweus, 1996), was used to measure the self-reported frequency of bullying. Prior to responding, the participants were provided with the definition of bullying (Olweus, 1996). Responses to the question *“How often have you bullied others at school in the last couple of months?”* were given on a 5-point scale (0 = not at all, 1 = only once or twice, 2 = two or three times a month, 3 = about once a week, and 4 = several times a week). The global item has been shown to be valid measure of bullying (Olweus & Limber, 2019).

### Analysis Plan

The aim was to test whether feeling guilty, ashamed, or sad mediated the association between perceived anti-bullying messages and intention to stop bullying. In all analyses, the teacher speaking in the video (male or female), gender of the participant, and self-reported frequency of bullying were controlled for, as they might be associated with students’ perceptions of the teacher message, as well as their emotions and intentions following the situation. First, we tested the main effects of perceived condemning of the bullying behavior and perceived empathy-raising on intention to stop bullying behavior (Model 1, Hypothesis 1). Second, we examined the effects of guilt, shame and sadness on intention to stop (Model 2, Hypothesis 2). Third, we examined the effects of the perceived messages on the emotions (Model 3, Hypothesis 3). Fourth, Models 1, 2 and 3 were combined to examine the mediating role of each emotion in the associations between the perceived messages and intention to stop (Model 4, Hypothesis 4). Finally, an exploratory analysis was conducted to test whether the effects were moderated by gender. The hypothesized mediators were allowed to correlate. Analyses were conducted using *M*plus 8.3 (Muthén & Muthén, 1998–2023) and maximum likelihood estimation (ML). The significance of the indirect effects of perceived messages on intention to stop bullying via the emotions was tested with bias-corrected bootstrapped confidence intervals (95%) for indirect and direct effects, using 10,000 bootstrap draws. Indirect and direct effects were considered significant when zero was outside of the range of confidence intervals (Preacher & Hayes, 2004). Missing data were handled using full information maximum likelihood estimation (FIML). The differences between the test groups were accounted for by using the COMPLEX option in *M*plus which corrects for distortions in standard error estimates caused by the clustering of observations (i.e., between-level variations). Test group was chosen as the clustering variable in order to control for situational factors in the testing session that might make the responses of students within test groups more similar to each other. The ICC for intention to stop bullying indicated that 4% of the total variance was due to differences between test groups.

## Results

### Effects of Perceived Messages and Emotions on Intention to Stop Bullying

Correlations and descriptive statistics for the study variables are presented in Table 1. In Model 1, the main effects of perceived messages on intention to stop bullying were tested (see Table 2). The model explained 32.6% of the variance in intention to stop bullying. Both perceived condemning of the bullying behavior and perceived empathy-raising positively predicted students’ intention to stop bullying. The effects of feeling guilty, ashamed and sad on intention to stop bullying were tested in Model 2 (see Table 2). The model explained 40.9% of the variance in intention to stop bullying. Guilt and sadness positively predicted students' intention to stop bullying, whereas shame had no significant effect.

**Table 1:** Correlations and descriptive statistics of study variables

Variable	*1*	*2*	*3*	*4*	*5*	*6*	*7*	*8*	*9*
Intention to stop bullying	-								
Perceived condemning	.09	-							
Perceived empathy-raising	.29***	-.28***	-						
Boy (student)	-.21***	-.08	.08	-					
Male (teacher)	-.10	-.04	.05	-.05	-				
Frequency of bullying	-.40***	.06	-.02	.12*	.05	-			
Guilt	.54***	.23***	.11	-.18**	-.08	-.25***	-		
Shame	.48***	.25***	.09	-.21***	-.10	-.16**	.79***	-	
Sadness	.45***	.09	.19**	-.20***	.02	-.19**	.54***	.55***	-
*M*	4.15	3.60	3.51	0.53	0.48	0.13	3.89	3.67	2.99
*SD*	0.91	1.45	1.16			0.41	1.39	1.45	1.52
Min	0.83	0.00	0.00			0.00	0.00	0.00	0.00
Max	5.00	5.00	5.00			3.00	5.00	5.00	5.00

*N* = 277. Correlations coefficients between binary variables are phi coefficients

***p <.001. **p <.01. *p <.05

**Table 2:** Effects of perceived messages and emotions on intention to stop bullying

	Model 1			Model 2		
Predictor	*b*	95% *CI*	*SE*	*b*	95% *CI*	*SE*
Boy (student)	**−0.32**	**[−0.47, −0.18]**	**0.08**	−0.12	[−0.30, 0.04]	0.09
Male (teacher)	−0.17	[−0.36, 0.04]	0.10	−0.12	[−0.26, 0.03]	0.07
Frequency of bullying	**−0.87**	**[−1.07, −0.60]**	**0.12**	**−0.59**	**[−0.82, −0.35]**	**0.12**
Perceived condemning	**0.13**	**[0.06, 0.21]**	**0.04**			
Perceived empathy-raising	**0.29**	**[0.20, 0.38]**	**0.05**			
Guilt				**0.19**	**[0.07, 0.35]**	**0.07**
Shame				0.06	[−0.06, 0.18]	0.06
Sadness				**0.11**	**[0.03, 0.17]**	**0.04**
R^2^	**0.326**		**0.06**	**0.409**		**0.06**

*N* = 277. Significant associations are in bold

### Effects of Perceived Messages on Emotions

Model 3 examined the effects of perceived messages on feelings of guilt, shame, and sadness (see Table 3). The predictors explained 18.6% of the variance in guilt, 16% of the variance in shame and 13% of the variance in sadness. Perceived condemning of bullying positively predicted feelings of guilt and shame but had no significant effect on sadness, whereas perceived empathy-raising was positively associated with all three emotions.

**Table 3:** Effects of perceived messages on feeling guilty, ashamed and sad

	Model 3								
	Guilt			Shame			Sadness		
Predictor	*b*	95% *CI*	*SE*	*b*	95% *CI*	*SE*	*b*	*95% CI*	*SE*
Boy (student)	**−0.42**	**[−0.65, −0.14]**	**0.13**	**−0.55**	**[−0.81, −0.30]**	**0.13**	**−0.57**	**[−0.87, −0.25]**	**0.16**
Male (teacher)	−0.20	[−0.54, 0.8]	0.16	−0.28	[−0.62, 0.04]	0.17	0.06	[−0.27, 0.36]	0.17
Frequency of bullying	**−0.84**	**[−1.21, −0.28]**	**0.24**	−0.52	[−1.13, 0.08]	0.31	**−0.67**	**[−1.11, −0.19]**	**0.23**
Perceived condemning	**0.28**	**[0.17, 0.40]**	**0.06**	**0.29**	**[0.16, 0.42]**	**0.07**	0.16	[−0.02, 0.33]	0.09
Perceived empathy-raising	**0.24**	**[0.08, 0.41]**	**0.09**	**0.24**	**[0.05, 0.42]**	**0.10**	**0.31**	**[0.17, 0.48]**	**0.08**
R^2^	**0.186**		**0.06**	**0.160**		**0.05**	**0.130**		**0.04**

*N* = 277. Significant associations are in bold

### Indirect Effects of Perceived Messages on Intention to Stop Bullying

**Fig. 1:**
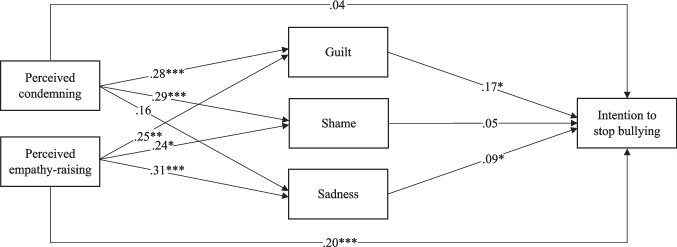
Direct and indirect effects of perceived messages on students’ intention to stop bullying, controlling for gender of the student, gender of the teacher, and self-reported frequency of bullying

Indirect effects of perceived messages on intention to stop bullying via emotions were examined in Model 4, which was built by combining Models 1, 2 and 3. The results are presented in Table 4 and Fig. 1 depicts the full model with both direct and indirect effects. There were significant indirect effects of both perceived condemning of the bullying behavior and perceived empathy-raising on intention to stop bullying via feelings of guilt and sadness. However, the indirect effects of perceived condemning of the bullying behavior and empathy-raising via feelings of shame were not significant. Besides these indirect effects, perceived empathy-raising (but not perceived condemning) continued to have a direct effect on students’ intention to stop bullying. Guilt and sadness positively predicted intention to stop bullying, whereas being a boy and self-reported frequency of bullying were negatively associated with intention to stop.

**Table 4:** Direct effects of messages and emotions, and indirect effects of perceived messages via emotions on intention to stop bullying

	Model 4		
Variable	*b*	95% *CI*	*SE*
Boy (student)	**−0.17**	**[−0.33, −0.03]**	**0.08**
Male (teacher)	−0.14	[−0.29, 0.05]	0.08
Frequency of bullying	**−0.63**	**[−0.83, −0.40]**	**0.11**
Perceived condemning	0.04	[−0.03, 0.12]	0.04
Perceived empathy-raising	**0.20**	**[0.10, 0.28]**	**0.04**
Guilt	**0.17**	**[0.05, 0.32]**	**0.07**
Shame	0.05	[−0.07, 0.17]	0.06
Sadness	**0.09**	**[0.02, 0.15]**	**0.04**
Indirect effects via guilt			
Perceived condemning	**0.05**	**[0.02, 0.10]**	**0.02**
Perceived empathy-raising	**0.04**	**[0.01, 0.12]**	**0.03**
Indirect effects via shame			
Perceived condemning	0.02	[−0.02, 0.06]	0.02
Perceived empathy-raising	0.01	[−0.02, 0.05]	0.02
Indirect effect via sadness			
Perceived condemning	**0.01**	**[0.00, 0.04]**	**0.01**
Perceived empathy-raising	**0.03**	**[0.01, 0.06]**	**0.01**
R^2^	**.464**		**.06**

*N* = 277. Significant associations are in bold

### Comparison Between Girls and Boys

As an additional exploratory analysis, moderation by gender was tested. Model fit comparison of the full mediation models indicated that the constrained model, in which parameters were held equal across gender, had slightly better fit (Akaike Information Criterion, AIC = 5649.47; Bayesian Information Criterion, BIC = 5928.24) than the freely estimated model (AIC = 5656.50; BIC = 5975.01). This suggests that the direct and indirect effects of perceived messages on intention to stop bullying did not differ significantly between boys and girls. Additionally, a Wald test of parameter constraints showed that the direct effects of perceived condemning of bullying behavior (*b* = −0.08, *p* =.186) and empathy-raising (*b* = −0.10, *p* =.346) on intention to stop bullying did not significantly differ between girls and boys. Similarly, the indirect effects of perceived condemning did not differ by gender via guilt (*b* = 0.03, *p* =.546), shame (*b* = −0.02, *p* =.625) and sadness (*b* = 0.02, *p* =.357), nor did the indirect effects of perceived empathy-raising via guilt (*b* = 0.02, *p* =.662), shame (*b* = −0.03, *p* =.463) and sadness (*b* = 0.01, *p* =.833).

## Discussion

To improve the effectiveness of targeted interventions, it is crucial to understand how they produce their effects. However, no study to date has examined the mechanisms through which different intervention strategies can stop the bullying. Using video vignettes depicting a teacher talking to the student (the participant) after they had (hypothetically) bullied a peer, the present study shed light on some of the psychological mechanisms through which the different messages – condemning of the bullying behavior and empathy-raising – lead to intention to stop bullying in a normative sample of youth.

First, we examined whether and how perceived condemning of the bullying behavior and perceived empathy arousal were associated with students’ intention to stop their bullying behavior. Supporting our hypothesis, both perceived condemning of the bullying behavior and empathy-raising positively predicted students’ intention to stop their bullying behavior. In line with previous studies, these findings provide further evidence that both strategies can be effective in stopping bullying (Garandeau et al., 2014, 2016; Johander et al., 2021, 2022).

Second, we examined whether feelings of guilt, shame, and sadness would be associated with the intention to stop. As expected, both guilt and sadness – but not shame – were positively associated with students’ intention to stop bullying. These results align with findings that empathic concern encourages prosocial and reparative behaviors (Eisenberg et al., 1989; Schumann & Dragotta, 2021), as well as with theoretical predictions (e.g., Tangney et al., 2007) and previous research showing that guilt is more likely than shame to lead to socially beneficial and corrective behaviors (e.g., Roos et al., 2014).

Third, we examined whether the perceptions of the messages would be associated with feelings of guilt, shame and sadness. As expected, perceived condemning of bullying behavior and perceived empathy-raising had a positive effect on students' feelings of guilt. It is possible that explicitly assigning responsibility to the perpetrator and condemning their behavior heightens individuals’ awareness of the immorality of their actions, eliciting guilt. Further, focusing the student’s attention on the victim's suffering may elicit guilt, as they recognize their own role in causing it. Although we expected only perceived condemning of the bullying behavior to be positively associated with shame, both condemning and empathy-arousal positively affected students’ feelings of shame. It seems that, although the condemning focuses on the behavior and not on the perpetrator, it has potential to arouse shame, as we expected. It is known that guilt and shame frequently co-occur (e.g., Hendriks et al., 2022). In addition, adolescents may have internalized the condemnation of their actions to reflect on them as a person (Tangney & Dearing, 2002). Condemning the behavior may also have reinforced the perception that their actions failed to meet social and moral expectations, leading to feelings of shame (Fessler, 2007). Perceived empathy-raising was also positively associated with feelings of shame. Research has shown that people differ in their dispositional tendency to experience shame, i.e., in their shame-proneness (Carpenter et al., 2016; Tangney et al., 2007). Thus, it is possible that regardless of what is said in the intervention discussion, it may elicit shame in some adolescents. As shame has been theorized to relate to one’s sense of self-worth (Lewis, 1971, as cited in Tangney et al., 2007), interventions should focus on condemning the behavior rather than induce shame by blaming the perpetrators and humiliating them. Finally, as expected, perceived empathy-raising positively affected students’ feelings of sadness, whereas perceived condemning did not. It seems that describing the targeted peer’s distressing situation, and how they must be feeling because of it, can arouse empathic concern in students.

Fourth, we examined the mediating role of emotions in the effects of perceived teacher messages on intention to stop bullying. We found that feelings of guilt and sadness mediated the effects of both, perceived condemning of the bullying behavior and perceived empathy-raising. However, as perceived condemning of the bullying behavior was not significantly associated with feelings of sadness, it seems that the effects of perceived condemning of the bullying behavior on intention to stop occur primarily through feelings of guilt.

Regarding the control variables, two interesting results emerged. First, boys reported less guilt, shame, and sadness than girls, a finding that also aligns with previous studies. Girls are generally more likely than boys to report feelings of guilt and shame, especially from adolescence onward (for meta-analysis, see Else-Quest et al., 2012). It has been proposed that the socialization process encourages girls more strongly than boys toward interpersonal connectedness and sensitivity (Benetti-McQuoid & Bursik, 2005; Bybee, 1998), as well as the adoption of expressive traits, such as empathy (Hoffman, 1977). It is possible that deviating from these expectations (e.g., bullying behavior) results in higher feelings of guilt and shame in girls. In addition, girls tend to score higher than boys on trait empathic concern (Trentini et al., 2022; Van der Graaff et al., 2014), which has been positively associated with state empathic sadness (Van der Graaff et al., 2016). Thus, girls' higher tendency to experience empathic concern might result in higher feelings of sadness in girls compared to boys. It is also possible that these observed gender differences reflect differences in reporting emotions rather than experiencing them.

Second, self-reported frequency of bullying negatively predicted feelings of guilt and sadness. Thus, the more a student reported having recently engaged in bullying behavior in real life, the less guilt and sadness they believed they would feel after this type of targeted intervention. This is in line with previous studies showing that bullying correlates negatively with feelings of guilt and empathy (Mazzone et al., 2016; Mitsopoulou & Giovazolias, 2015). However, contrary to previous research showing that bullying also correlates negatively with shame (Menesini & Camodeca, 2008), bullying frequency was unrelated to feelings of shame in the current study. Thus, our participants anticipated experiencing similar levels of shame following this type of intervention, regardless of whether they had previously engaged in bullying or not. As shame is tied to one’s sense of self (e.g., Tangney & Dearing, 2002), it is possible that bullying students feel shame when confronted, not necessarily because of their actions, but rather due to its potential negative impact on their self-perceptions or social standing.

Finally, we explored whether gender moderated the direct effects of the perceived messages on intention to stop bullying, as well as the indirect effects via each emotion. We found no significant moderation effects. Thus, although boys reported lower levels of guilt, shame and sadness, the results suggest that the emotional pathways though which perceived condemning of bullying behavior and empathy-raising affect the intention to stop bullying are similar across genders.

## Limitations

Since the study used video vignettes depicting an adult talking to a student who had been bullying others, both the students' bullying behavior and the targeted intervention were hypothetical. The students were instructed to imagine how they would feel and respond if they had been in such an intervention after engaging in bullying. Thus, the extent to which the results can be generalized to real-life situations remains unclear and should be addressed in future research.

Second, only 32 students in the sample reported having bullied others at school within the past few months, which did not allow us to test our hypotheses in a subsample of bullying perpetrators. However, the more a student reported bullying others in real life, the less guilt and sadness they believed to feel after hearing the teacher’s message. Thus, the results could be different in a sample consisting only of students who engage in high levels of bullying in real life. On the other hand, bullying is a group phenomenon in which many students engage in mean behaviors to varying degrees and in different roles (Salmivalli, 2010). Moreover, some students may have engaged in bullying outside the time frame specified in the present study (the past couple of months), even if they did not report doing so during this period. It is also known that students who engage in high levels of bullying tend to underreport their behavior (Garandeau et al., 2025). Therefore, it is likely that there were students in our sample who did not disclose their bullying behavior. Overall, the intervention scenario can be considered a situation many students in our normative sample could encounter in real life. Moreover, if underreporting of bullying occurred, it may have weakened (rather than strengthened) the observed associations, as students who reported bullying expressed lower levels of guilt and sadness. Future studies on students’ perceptions of teacher interventions could assess bullying behaviors using multiple items depicting mean behaviors rather than asking about “bullying” or by utilizing peer reports instead of self-reports.

Third, although the exact number of students in each participating classroom is unknown, based on the average classroom size in Finnish schools, we estimate the participation rate to be around 40%. Thus, on average, less than half of the students in participating classrooms took part in the study. Consequently, the sample may not be fully representative of the target population, which could limit the generalizability of the findings.

Fourth, the study did not take students' psychological characteristics into consideration. Individual characteristics are likely to influence how the messages are perceived, as well as the emotions they evoke. For instance, empathy and callous-unemotional traits have been shown to be related to bullying behavior (Geel et al., 2017; Mitsopoulou & Giovazolias, 2015), as well as to the intention to stop bullying after an intervention (Johander et al., 2022). Examining the role of such characteristics in future studies would provide further insight. Finally, due to the COVID-19 pandemic and the resulting lockdown in Finland during the spring of 2020, some students took part in the study online, which led to reduced control over the study conditions.

## Conclusions

Perceived condemning of the bullying behavior and empathy-arousal both increase students’ intention to stop bullying after a teacher’s targeted intervention. The effects of perceived empathy arousal on intention to stop were mediated by both feelings of sadness and guilt, and the effects of perceived condemning of the bullying behavior were mostly mediated by guilt. Shame was unrelated to intention to stop, and did not mediate the effects of the perceptions. These findings suggest that anti-bullying interventions should integrate strategies that promote guilt and empathy, while avoiding the elicitation of shame. This implies focusing on condemning the behavior rather than making students feel inherently flawed and inferior by blaming them, as shame is unlikely to be effective in reducing bullying. Furthermore, research has shown that even when bullying prevention programs provide clear guidelines on how to intervene in bullying, school personnel do not always implement the approaches as intended; instead, they often make their own adaptations (Johander et al., 2021). Therefore, rather than providing teachers with rigid instructions on what to say and do in intervention discussions, programs should focus on increasing their understanding of the mechanisms behind the effectiveness of different approaches. Such understanding may, in turn, make teachers more likely to implement these strategies effectively in real-life interactions with students.

## Data Availability

The fully anonymized dataset is available from the corresponding author upon reasonable request.
